# On the Modularity of the Intrinsic Flexibility of the *µ* Opioid Receptor: A Computational Study

**DOI:** 10.1371/journal.pone.0115856

**Published:** 2014-12-30

**Authors:** Mathieu Fossépré, Laurence Leherte, Aatto Laaksonen, Daniel P. Vercauteren

**Affiliations:** 1 Laboratoire de Physico-Chimie Informatique, Unité de Chimie Physique Théorique et Structurale, University of Namur (UNamur), Namur, Belgium; 2 Arrhenius Laboratory, Division of Physical Chemistry, Stockholm University, Stockholm, Sweden; 3 Namur Medicine and Drug Innovation Center (NAMEDIC), University of Namur (UNamur), Namur, Belgium; 4 Stellenbosch Institute of Advanced Study (STIAS), Wallenberg Research Centre at Stellenbosch University, Stellenbosch, South Africa; Oak Ridge National Laboratory, United States of America

## Abstract

The *µ* opioid receptor (*µ*OR), the principal target to control pain, belongs to the G protein-coupled receptors (GPCRs) family, one of the most highlighted protein families due to their importance as therapeutic targets. The conformational flexibility of GPCRs is one of their essential characteristics as they take part in ligand recognition and subsequent activation or inactivation mechanisms. It is assessed that the intrinsic mechanical properties of the *µ*OR, more specifically its particular flexibility behavior, would facilitate the accomplishment of specific biological functions, at least in their first steps, even in the absence of a ligand or any chemical species usually present in its biological environment. The study of the mechanical properties of the *µ*OR would thus bring some indications regarding the highly efficient ability of the *µ*OR to transduce cellular message. We therefore investigate the intrinsic flexibility of the *µ*OR in its apo-form using all-atom Molecular Dynamics simulations at the sub-microsecond time scale. We particularly consider the *µ*OR embedded in a simplified membrane model without specific ions, particular lipids, such as cholesterol moieties, or any other chemical species that could affect the flexibility of the *µ*OR. Our analyses highlighted an important local effect due to the various bendability of the helices resulting in a diversity of shape and volume sizes adopted by the *µ*OR binding site. Such property explains why the *µ*OR can interact with ligands presenting highly diverse structural geometry. By investigating the topology of the *µ*OR binding site, a conformational global effect is depicted: the correlation between the motional modes of the extra- and intracellular parts of *µ*OR on one hand, along with a clear rigidity of the central *µ*OR domain on the other hand. Our results show how the modularity of the *µ*OR flexibility is related to its pre-ability to activate and to present a basal activity.

## Introduction

**Figure 1:**
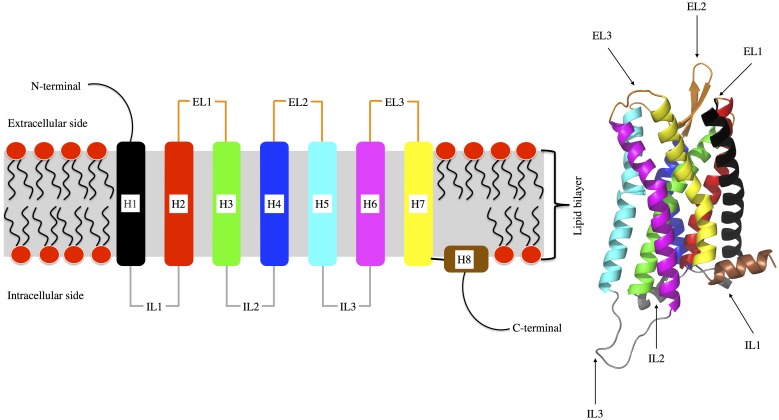
Left: Schematic view of the overall structure of a G protein-coupled receptor (GPCR), with depiction of the connectivity of the intracellular (IL) and extracellular (EL) loops between helices (H). Right: 3D structure of the reconstructed *µ*OR structure (see “Methods”). Color codes of ILs, ELs, and Hs are identical in both left and right pictures.

G-protein coupled receptors (GPCRs) represent the most important protein superfamily of cell surface receptors as the target of almost 50% of approved drugs in the market [1]. They are implicated in a variety of cellular signals as responses to neurotransmitters or hormones, but also to odorant molecules or to light in the case of rhodopsin. Structurally, GPCRs consist of a bundle of seven transmembrane *α*-helices (H1–H7), a soluble *α*-helix (H8) in the intracellular domain, three extracellular loops (EL1–EL3), a N-terminal domain, and three intracellular loops (IL1–IL3) along with a C-terminal domain in the intracellular side (Fig. 1). It took seven years until the second resolved GPCR crystallographic structure [2], *i.e.*, human *β*2 adrenergic receptor, appeared in literature after the first one was reported, *i.e.*, bovine rhodopsin in 2000 [3]. Interestingly, the last few years have seen a dramatic increase in the release of new crystal structures of various GPCRs in the Protein Data Bank (PDB) [4], [5].

Among the recently available GPCRs in the PDB, the three subtypes of opioid receptors (ORs), *i.e.*, *µ*, *δ*, and *κ* [6]–[9], are particularly important for the medical community as their opioid alkaloids like morphine are the most effective analgesics for the treatment of pain [10]. More specifically, *µ*OR is the receptor morphine interacts the most among the three OR subtypes [11]. For this reason, *µ*OR is the principal target in general anesthesia by administration of compounds such as fentanyl and its derivatives, even if still limited by several side effects like addiction and dependence or tolerance [12], [13]. Hence, many studies have already stressed the need and importance to develop safer and more effective therapeutic ligands targeting *µ*OR [14], [15].

Although the recent rapid progress in GPCR crystallization represents a milestone for the pharmaceutical research, each of the three-dimensional (3D) structures now available in PDB represents a single low energy conformation without any dynamical information. Besides, most of the crystallized GPCRs were often resolved at a rather low temperature compared to physiological temperatures, or were obtained after fusing the protein with the N-terminal T4 lysozyme to facilitate the crystallization [16], both conditions imposing possible structural constraints on the receptor conformation.

The sudden rise of new crystal structures has provided a rich source of information for subsequent modeling studies [17], [18] to investigate various GPCRs related biomedical applications such as new potent ligand discoveries [19] as well as the elucidation of important dimerization/oligomerization mechanisms [20].

Hence, conformational structure and dynamics investigations of GPCRs, with theoretical methods such as Molecular Dynamics (MD) simulations in a biologically relevant environment, are of a great interest to understand how the dynamical properties of GPCRs are related to their biological functions [21], [22]. In order to fully understand the multiple biological roles of GPCRs, it is necessary to understand the conformational complexity of the highly flexible GPCRs exhibiting a wide spectrum of conformations that can interconvert throughout highly complex equilibrium [23]–[26]. Even after a GPCR forms a complex with an agonist, it remains in an equilibrium involving several different conformations of its possible active states [27] as well as many different conformations can play a role in the signaling mechanism pathways [28]. Furthermore, many factors such as the role of a dimer- and/or oligomer-state [29], the biological environment, *i.e.*, the pH [30], [31], the lipid membrane composition [32], [33], the ion concentrations on possible voltage-dependent mechanisms [34], all complicate the description of the GPCR dynamics. Evidently, the emerged picture of GPCRs as highly complex ensembles of conformations is directly related to their intrinsic flexibility properties. Recent studies show that GPCRs can induce an activation signal even in the absence of endogenous agonists [35], which is called the basal activity of GPCRs and most notably for *µ*OR as its basal activity is potentially playing a role in addiction and in narcotic dependence [36], [37]. A detailed analysis of the important physiological role related to the intrinsic flexibility of the *µ*OR is clearly thus needed.

Theoretical studies on the role of the intrinsic flexibility in GPCRs have already demonstrated that considering a GPCR in its apo-form can help to better understand its dynamics and their pre-ability to undergo conformational changes related to their activation mechanism. For instance, MD simulations of the adenosine A2A receptor showed that the H3 and H4 helices are remarkably more stable compared to H1, H2, H5, H6, and H7 [38] and revealed the essential role of several conserved residues for both A2A and A2B adenosine receptors [39]. To assess the importance of the flexibility properties of the *µ*OR that guide their basal activity, we focus here on its dynamics at the monomer state to depict the functional role of the *µ*OR apo-form dynamics. Although MD simulations of the *µ*OR complexed with antagonists or agonists have recently been performed [40], MD simulations of the *µ*OR apo-form based on the recent crystal structure determination of *µ*OR are, to our knowledge, absent in the literature. Only one MD study of the ligand-free *µ*OR has been recently published [41]. In their paper, the authors study the influence of the water and the ions in the binding of ligands but still not deal with the intrinsic flexibility properties and their biological role without any structural bias due to bilayer components.

In the present paper, we will focus on the underlying functional role of the *µ*OR flexibility properties by performing sub-microsecond all-atom (AA) MD simulations without, at purpose, any ligand or any molecular moiety that could modify the intrinsic flexibility properties of the *µ*OR. In the *Methods* section, we will describe the tools used to investigate the intrinsic flexibility of *µ*OR. In *Results*, we will show how the dynamical properties of *µ*OR are related to the communication and interdependence between the domain regions. Our studies will hence allow depicting how the *µ*OR dynamical properties are associated with its predisposition to facilitate biological functions as its basal activity. We will next consider the modularity and the subsequent coupling between the *µ*OR domains as a way to consider the GPCR structures now becoming popular in literature [42], [43].

## Methods

### A. Molecular dynamics simulations

The 3D structure of the *µ*OR was built from the coordinates obtained by X-ray diffraction structure [6] that are available in the PDB (ID: 4DKL). *µ*OR was crystallized at a resolution of 2.80 Å with an antagonist covalently bounded, *i.e.*, *β-*funaltrexamine, water molecules, sulfate ions, chloride ions, a cholesterol molecule, a pentaethylene glycol molecule, and a 1-monooleoyl-rac-glycerol molecule. To study the intrinsic flexibility of the *µ*OR, all of these species were removed to conserve only the *µ*OR structure in the Molecular Dynamics (MD) simulation. Also the T4 lysozyme structure, inserted between H5 and H6 to enhance crystallogenesis of the *µ*OR, was removed and replaced with the corresponding six missing residues of the loop IL3, *i.e.*, M264-L265-S266-G267-S268-K269. The latter sequence comes from the *µ*OR primary sequence of the mouse from which it was crystallized, *i.e.*, available online in the UniProt database (ID: P42866) [44]. The missing loop IL3 was constructed with the FALC-Loop webserver, a protein loop modeling method based on fragment assembly, developed by Ko *et al.* [45]. Among the 2,000 models generated, the best ranking model according to the FALC-Loop energy scoring, *i.e.*, the DFIRE energy, was selected as a starting structure.

The *µ*OR 3D structure, 288 residues for 4,728 atoms, was then inserted into a model bilayer composed of 259 1-palmitoyl-2-oleoylphosphatidylcholine (POPC) molecules, 17,199 water molecules, and 14 Cl^−^ for neutralizing the system, for a total of 88,337 atoms. Our system consists therefore to a simplified model of the membrane with a one-component lipid, an environment that has already been proven to be sufficiently valuable to study other GPCRs dynamical properties [46], [47].

In the membrane model, cholesterol and sodium ions were not, as mentioned before in the *Introduction*, considered to let specifically the *µ*OR structure evolve during the MD simulation thanks to its flexibility properties only and not to any structural constraint due to components of the membrane environment. Other studies have highlighted the specific role of these membrane components [48], [49], but important GPCRs mechanisms have also been studied with simplified model membranes [44], [45], similar to the one used here.

While building the topology files using VMD [50], we paid a special attention to the atom type attribution to the sulfur atoms of residues C140 and C217, those two ones bound together to form the unique disulfide bridge of the crystal structure of the *µ*OR between H3 and EL2. The latter should effectively be crucial for the overall dynamics characteristics of the *µ*OR as this disulfide bridge will retain EL2 closer to H3.

The MD simulations were performed using the NAMD 2.8 package [51] with the CHARMM22-CMAP [52] force field (FF) for the protein and CHARMM 36 FF for the lipids [53]. The total energy was first minimized in 1,000 steps using the conjugate gradient to remove contacts that could lead to high energy values. To provide the fluidity in the tails of the lipids, a first MD run of 1 ns, the melting lipid step in which the whole system except lipids is frozen, was performed in the constant volume-temperature (NVT) ensemble using Langevin dynamics [54], [55]. The temperature and the box size were fixed at 310.0 K and at 98×98×103 Å, respectively. A time step of 2 fs was used to integrate the equations of motion as covalent bonds involving hydrogen atoms were fixed by the SHAKE algorithm [56]. Coulomb interactions were computed with the particle-mesh Ewald (PME) summation method [57] with a grid of 100×100×105 Å, *i.e.*, at least one grid point per Å in each direction in such a way that the number of grid points is a multiple of two, three, or five. Van der Waals interactions by Lennard-Jones potential were smoothed from 10.0 to 12.0 Å by a switching function as introduced by Steinbach and Brooks [58]. The non-bonded pair list was generated until 13.5 Å. Periodic boundary conditions were used in the evaluation of the non-bonded interactions.

To allow rearrangement of the ion-water-lipid environment around the receptor, the rest of the system, *i.e.*, water molecules and ions, were then relaxed in a pre-equilibration stage by performing an equilibration of 1,000 steps followed by a 2 ns MD simulation with identical parameters as in the melting lipid stage except that, now, only the receptor coordinates were constrained in the NPT ensemble, with the pressure maintained at a value of 1.0 atm and the temperature at 310.0 K with the Langevin integrator. The final coordinates were then submitted to a 25 ns equilibration stage without any position constraints on the system. Coordinates were saved every 2 ps. Finally, a production simulation of 0.5 *µ*s was carried out, *i.e.,* a unique but sufficient long simulation to extract statistical reliable data coming from the *µ*OR structure fluctuations.

### B. Analysis of the Molecular Dynamics simulations

The analysis of the MD results regarding the protein flexibility was performed using the software EUCB [59]. We mainly considered the Root Mean Square Deviations (RMSD), Root Mean Square Fluctuations (RMSF), angles, and dihedral angles along the receptor backbone as reported in Results. The Radius of Gyration (RG) was evaluated with the software Carma, another stand-alone MD analysis tool adapted for the DCD/PSF format used in NAMD [60]. The Ramachandran map was plotted with the RAMPAGE webserver [61]. Secondary structure assessment was performed with the built-in plugin *Timeline* of VMD [50]; the corresponding pictures were generated by an in-house Gnuplot script [62].

Flexibility and geometry analyses of the *α*-helices were performed with BENDIX [63], an extension plugin of VMD. In *Results*, the coarse-grained abstraction of the *α*-helices geometry is called a “BENDIX” representation. The BENDIX angle here corresponds to the maximum angle observed along the considered *α*-helix, independently of its position along the helix.

The volume of the binding site was evaluated with the POCASA webserver [64], POCASA standing for POcket-CAvity Search Application. It is used for the prediction of ligand binding sites of proteins and is based on the exploration of a 3D grid size of points with a probe sphere. POCASA allows the user to select the probe radius and the size of the unit grid, *i.e.*, 2.0 Å and 1.0 Å, respectively, in our case. As several pockets can be found for the target protein, the user has to select how many ranked pockets will be considered for analysis; five in our case. POCASA outputs the volume and the ranking of each pocket of the protein.

To modularize the *µ*OR structure according to its flexibility properties, two approaches were considered, *i.e.*, (i) the Geometrically Stable Substructures program (GeoStaS) [65] and (ii) the dynamical network analysis strategy introduced by Sethi and coworkers [66], [67]. Regarding GeoStaS, the first step consists of searching for similarities between motions of groups of atoms. The input of GeoStaS is a set of conformations generated by the MD simulation. The basic assumption of the algorithm is that correlations between parts of a molecular system are observed if there are similar motions between those corresponding parts. The particularity of GeoStaS is to produce a trajectory for each atom of the system, and not only regarding the global conformations. The algorithm evaluates the similarity between the trajectories. Usually, a similar mode of motion of two entities, or two atoms, is detected through an evaluation of the ensemble of 3N coordinate vectors available in the MD trajectory. At the end of the process, a cross-correlation matrix is obtained. However, such a method does not allow detecting properly rotation motions as demonstrated by Romanowska *et al.* [65]. Therefore and to optimize their correlation motions, all atom trajectories are compared by finding an isometry between them, *i.e*., translation plus rotation motions. A pairwise similarity matrix of atomic motions is hence created by the calculation of a geometric similarity of the trajectories using quaternion-based techniques. The second step is to iteratively cluster the atoms to form domains of the molecular system by calculating the smallest mutual distance such as described in [65]. Therefore, the user needs to provide the final number of clusters as a criterion to stop the algorithm. To critically deduce the final number of cluster, we used a plot of the distance between the merged clusters *versus* the iteration number of the clustering algorithm. A large change in the distance between clusters acts as a signal that a further merging between clusters is equivalent to group unrelated clusters. The resolution degree considered in our analysis was defined at the level of the C*α* atoms.

The second approach, the dynamical network analysis strategy, called “network approach” here below, is implemented as an extension plugin in VMD. As detailed in Sethi *et al.* [66], the resolution of the network describing the protein structure such as one edge corresponds to one residue located on the C*α* atom. Pairs of nodes are connected based on the covariance, and correlation between pairs of atoms are calculated with Carma from the MD trajectories. Correlation data are then used to generate the weights for the dynamical network describing the protein dynamics. The resulting network is thereafter submitted to the Girvan-Newman algorithm [68] to determine the community substructure, *i.e.*, the set of sub-networks that partition the original network. As described in [68], the Girvan-Newman algorithm is based on the measure of the betweenness centrality of the network of residues. The betweenness centrality of a node consists in the number of shortest paths built from all vertices to all others that pass through the given node. Hence, the centrality measure allows to detect the important connector-like residues considering the global structure of the network, without any bias on a maximal distance between pairs of nodes.

The ProDy package [69] was considered together with the Normal Mode Wizard (NMWiz) VMD plugin. ProDy/NMWiz allows performing essential dynamics analysis (EDA) [70] of an MD trajectory to extract the principal conformational changes [71]. We based our analysis on only the C*α* atoms of the *µ*OR structure to compute and visualize the normal modes out of a Principal Components Analysis (PCA) of the MD data.

## Results and Discussion

The stability of the *µ*OR structure modeled in a simplified membrane was validated according to the convergence of the Root Mean Square Deviation (RMSD) (S1 Fig.). The structural quality assessment was achieved through the Ramachandran plot of the last structures of the MD equilibration protocol (S2 Fig.). In the next sections, we analyze the 0.5 *µ*s production MD data, as described in *Methods*, to further investigate the dynamical behavior of *µ*OR. Our analyses are divided in a hierarchical way regarding the *µ*OR structure. In *Section A*, we show that the overall shape of the *µ*OR structure is well preserved at the sub-microsecond time scale. In *Section B*, the *µ*OR structure was considered as a set of seven entities corresponding to the helices. A bendability scale of the different helices is pointed out. Important consequences at the atomic scale on the binding site properties, *i.e.*, shape and volume, are demonstrated in *Section C*.

In *Section D*, the *µ*OR structure is modularized, according to data extracted from the MD simulation, with two different approaches. These latters, fitting well together, are discussed in terms of the basal activity of the *µ*OR and can explain why the signal transduction is highly efficient during the activation mechanism according to the intrinsic flexibility of *µ*OR. Finally, in *Section E*, essential dynamics analyses are performed and rationalized with the modularization techniques used in the previous sections.

### A. Overall dynamical properties of *µ*OR

**Figure 2:**
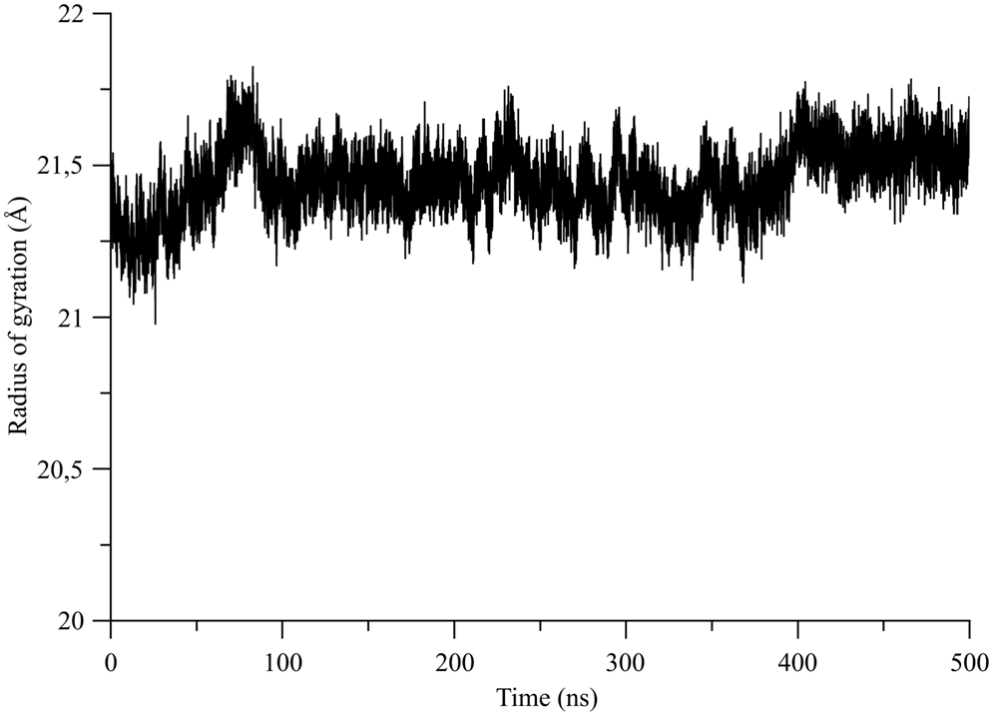
Radius of gyration of the *µ*OR structure along the 0.5 *µ*s all-atom MD simulation.

To understand how the global conformational state of *µ*OR is evolving with time, we analyzed various geometrical features of the *µ*OR structure along the 0.5 *µ*s all-atom MD production phase. First, the RMSD values following the 25 ns equilibration stage were plotted (S3 Fig.). The plateau reported in S3 Fig. implies no drastic change in the overall conformation of the *µ*OR structure during the production trajectory. Additionally, the standard deviation of the RMSD values between the starting frame of the equilibration and the generated conformations throughout the MD is very low, *i.e.*, below 0.24 Å. The radius of gyration, an indicator of the *µ*OR structure compactness, remains almost constant along the 0.5 *µ*s MD production stage (Fig. 2), with an average value of 21.45 Å and a SD of 0.11 Å. It indicates that the overall volume and thus the conformational state of *µ*OR is not evolving much, preserving therefore the global shape of the *µ*OR.

**Figure 3:**
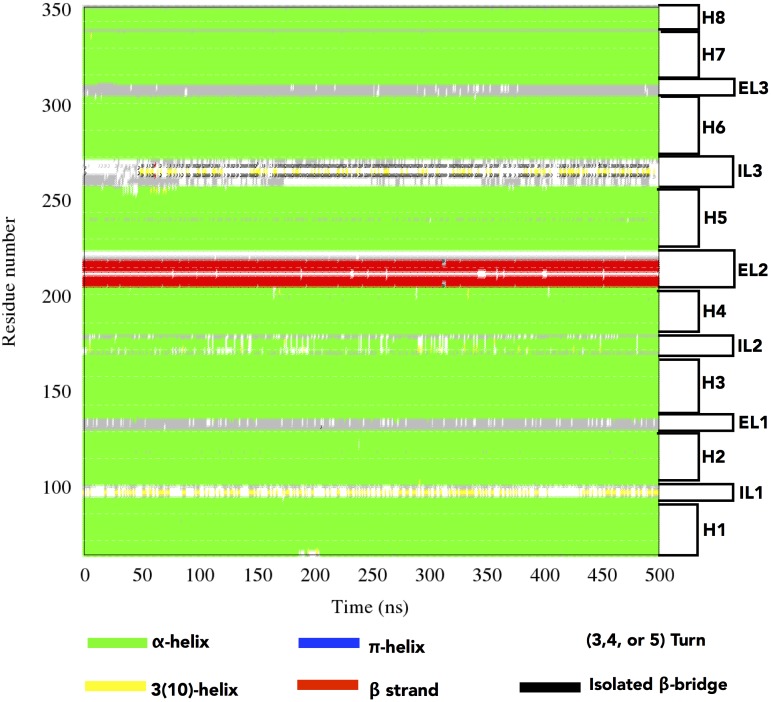
Identification of the secondary structure elements of the *µ*OR structure during the 0.5 *µ*s all-atom MD simulation. Secondary structures are classified according to the color code illustrated below. The regions for the different helices, ILs and ELs, with respect to the *µ*OR structure are depicted on the right side.

The secondary structure elements of the *µ*OR are well preserved throughout the simulation (Fig. 3), especially the seven *α*-helices, H1 to H7. Even the short coil of H5 involving the A240-F241-I242 residues, due to a small helix deformability coming from the presence of a proline at position 244 in the µOR sequence, is observed along the whole MD run. The short *α*-helix at the end of the receptor structure (H8), essential for the recognition between µOR and a G-protein, is preserved as well. The intracellular loop IL1 presents mainly a turn-type secondary structure but could be assigned to a coil or even a 3_10_ helix. The short *α*-helix of IL2 is mainly conserved, even if a coil-type conformation could be occasionally adopted. IL3 presents the most varying conformation in terms of secondary structure. It consists mainly of an alternating coil to turn conformation. We also noticed that some parts of IL3 can adopt an isolated bridge type secondary structure, meaning that longer MD runs over several µs can possibly fold IL3 in diverse conformational states. The extracellular loop EL1 switches between turn and coil; EL2 conserves its *β*-bridge (extended) whereas EL3 preserves a coil type.

**Figure 4:**
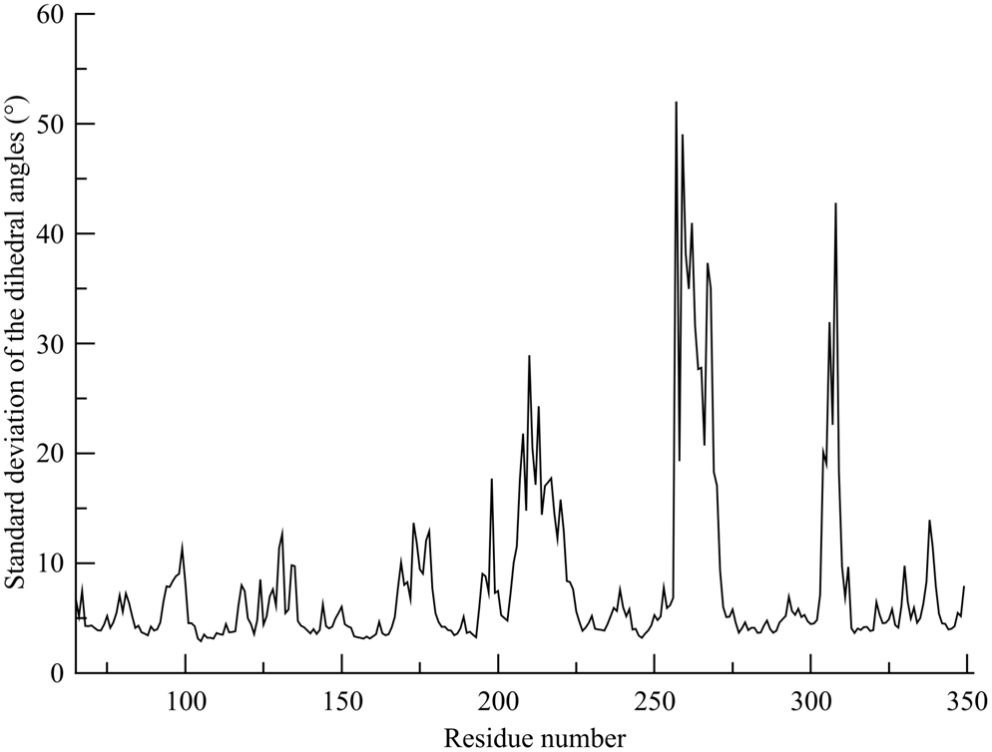
Standard deviation of the mean dihedral angles calculated from the 0.5 *µ*s all-atom MD production between four consecutives C*α* atoms along the *µ*OR backbone.

We next investigated the backbone geometrical properties for the different µOR domains to corroborate the high conservation of the secondary structural elements. The SD of the averaged dihedral angles between four consecutive C*α* atoms along the µOR backbone conformations generated during the MD production stage is presented in Fig. 4. The dihedral SDs averaged for the significant parts of the µOR are reported in Table 1. The small value of the dihedral SDs averaged over the entire receptor, *i.e.*, 7.9°, is in agreement with the conservative behavior of the secondary structure elements as noticed before. Fig. 4 illustrates the profile of the different degrees of torsional ability along the structure. The ILs and ELs are the most deformable parts of the µOR with mean SD values of 17.7 and 15.6°, respectively. More specifically, IL3 and EL2 with dihedral SD values of 28.1 and 16.0° are the most deformable loops between all ILs and ELs, respectively. The *α*-helices are less deformable with a mean dihedral angle SD of 5.5° for the bundle of the seven helices. H5 is the most deformable helix with a value of 6.8°. H2 and H3 are the most rigid ones with 5.0 and 4.5° values which are still close to the dihedral value of H5, denoting therefore a rather low difference of flexibility.

**Table 1:** Standard deviation (SD) of the mean dihedral angles computed from sequences of four consecutives C*α* atoms along the *µ*OR backbone from the 0.5 *µ*s all-atom MD simulation.

Element	SD of the dihedral angles (°)	Element	SD of the dihedral angles (°)
H1	5.09	EL1	8.04
H2	4.97	EL2	15.96
H3	4.48	EL3	22.05
H4	5.90	IL1	8.40
H5	6.75	IL2	9.73
H6	5.67	IL3	28.14
H7	5.88	ELs	15.60
H8	5.03	ILs	17.73

Therefore, we consider that the RMSD convergence, the stability of the gyration radius, the conservation of the secondary structure assignments, and the very small SDs around the mean backbone dihedral angles along the MD trajectory for the different receptor parts are all indicators that the overall shape of the structure is not strongly evolving with time. Nevertheless, these observations do not mean that µOR could be prevented from adopting different conformational states that might explain its flexibility-induced basal activity. In fact, the conformations of GPCRs are rather close in terms of their global shape as they are in many transmembrane proteins. The lipid environment of such proteins implies that large conformational changes in the bilayer would occur in general at a higher thermodynamic cost, compared to globular proteins in water solution. Indeed, a more viscous environment, as it is the case for the bilayer environment compared to water, implies a higher thermodynamic cost due to the needed reorganization following high conformational changes of a protein. Therefore the conformational differences are in general small, and thus have to be studied in terms of distances between sub-modules rather than in terms of the overall shape of the protein, as mentioned in *Introduction* for other GPCRs [42]–[43].

In the next section, we analyze the dynamical behavior of µOR and its possible biological significance in terms of the components of the receptor. The µOR structure is subdivided into seven entities corresponding to the helices. Our aim is to analyze the degree of flexibility of each of the helices, a mechanical feature of the µOR that could have important structural impact in the vicinity of the receptor where the orthosteric binding site is localized.

### B. Bendability of the *µ*OR helices

**Figure 5:**
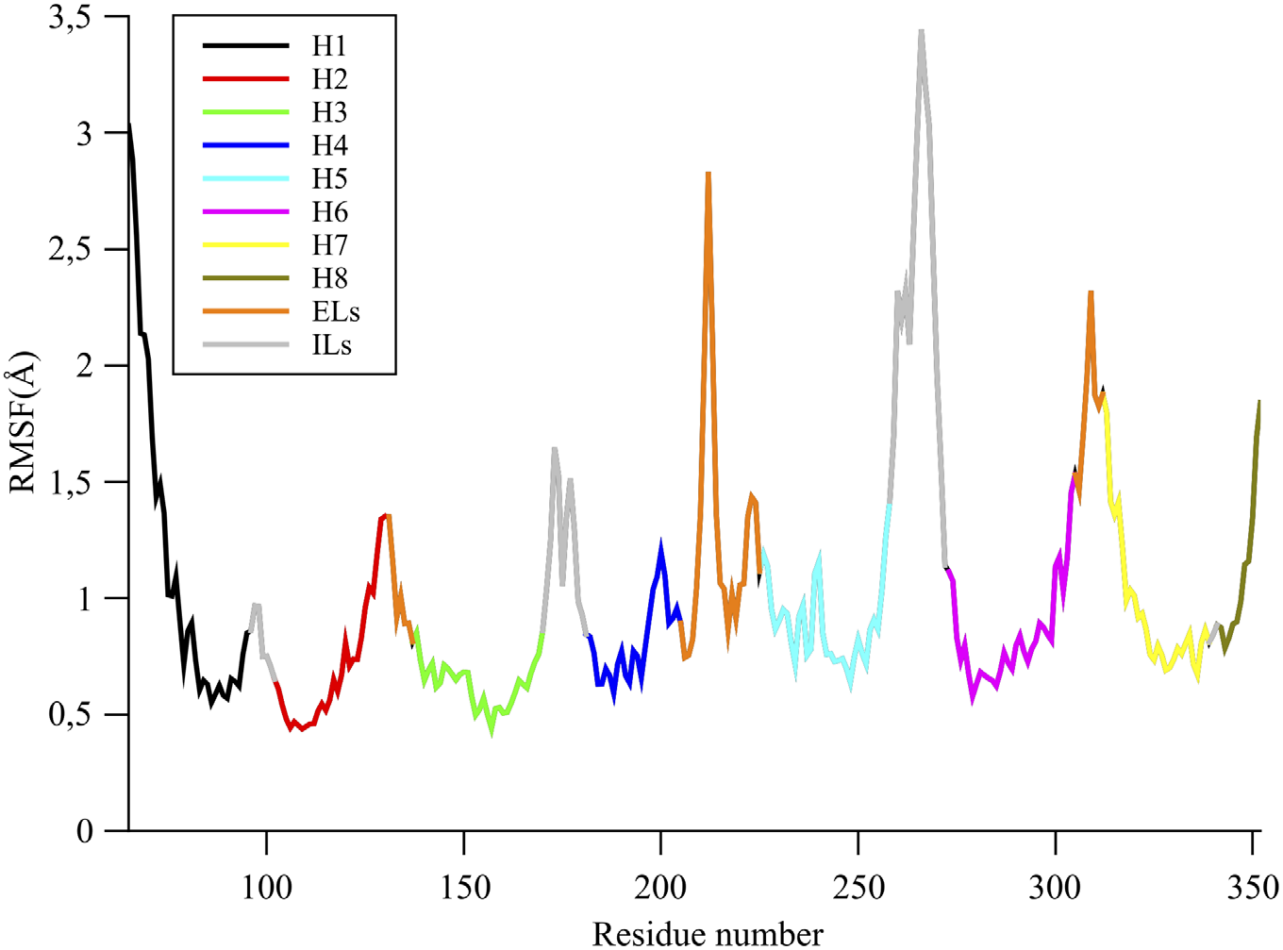
Root Mean Square Fluctuation (RMSF) of the C*α* atoms along the *µ*OR structure computed during the 0.5 *µ*s all-atom MD production. Color codes of ILs, ELs, and Hs are the same as in Figure 1.

To consider the bendability of the helices, we analyzed the Root Mean Square Fluctuations (RMSFs) along the backbone of the receptor structure in the 0.5 *µ*s all-atom MD simulation (Fig. 5). In Table 2, we present the average RMSF for the significant parts of the structure. The most flexible regions are, as expected, the ELs and ILs loops with a mean RMSF value of 1.34 and 1.66 Å. The *α*-helices are the most rigid parts. In this regard, it is interesting to notice that the degree of rigidity/flexibility of the seven *α*-helices can be very different. H2 and H3, the most rigid helices, show mean RMSF values of only 0.64 and 0.70 Å, respectively. We had already observed that H2 and H3 are also characterized by the lowest dihedral angle SDs. In contrast, H1 is the most flexible with a mean RMSF of 1.17 Å, which is almost twice as much as 0.64 Å for H3.

**Table 2:** Mean Root Mean Square Fluctuation (RMSF) computed for the C*α* atoms of the *µ*OR backbone from the 0.5 *µ*s all-atom MD simulation.

Element	RMSF (Å)	Element	RMSF (Å)
H1	1.17	EL1	1.00
H2	0.70	EL2	1.25
H3	0.64	EL3	1.82
H4	0.82	IL1	0.81
H5	0.90	IL2	1.19
H6	0.86	IL3	2.30
H7	0.97	ELs	1.34
H8	1.11	ILs	1.66

The RMSF analysis gives information about the ability of some parts of the receptor to deviate from the average structure of µOR during the MD simulation, but not about the capability for these domains to be intrinsically flexible and about their maximal deformation. We therefore investigated the backbone deformability for the different secondary structure elements. Indeed, even if the dihedral angle SDs are very weak, the values depict an average behavior and do not show that dihedral angles could momentarily divert significantly from their mean values due to a high deformability of some helices related to microstates of the µOR structure that could have an important biological role [23]–[25]. The capability of some helices to become distorted were calculated by considering the maximum angle values adopted by the µOR structures represented at the coarse-grained BENDIX resolution, as explained in Methods. The mean maximum BENDIX angles for each helices and their SDs are reported in Table 3. The order of bendability for the seven helices according to the mean maximum BENDIX angle is H7, H4, H6, H2, H3, H5, and H1, the last one presenting a mean maximum value still not negligible of 15.0°. It clearly indicates that the helices could be locally and momentarily highly deformed.

**Table 3:** Minimum, maximum, mean, and standard deviation (SD) values of the maximum helix angle value observed using the BENDIX representation for to the different helices of the *µ*OR structure from the 0.5 *µ*s all-atom MD simulation.

Helix	Mean BENDIXangle (°)	Minimum BENDIXangle (°)	Maximum BENDIX angle (°)	SD of the BENDIX angle (°)
H1	15.0	5.0	32.2	3.8
H2	27.8	9.1	53.6	6.1
H3	18.8	9.9	32.5	3.4
H4	32.5	13.9	49.6	4.8
H5	17.9	6.8	31.9	3.5
H6	28.5	14.2	42.6	3.8
H7	35.9	21.8	57.8	4.3

To illustrate the biological implications on how the deformability of the helices and the time dependence of their inter-distances affect the binding site of µOR, we analyze, in the following part, how the binding site shape of the µOR can evolve throughout time.

### C. Dynamical properties of the *µ*OR binding site

**Figure 6:**
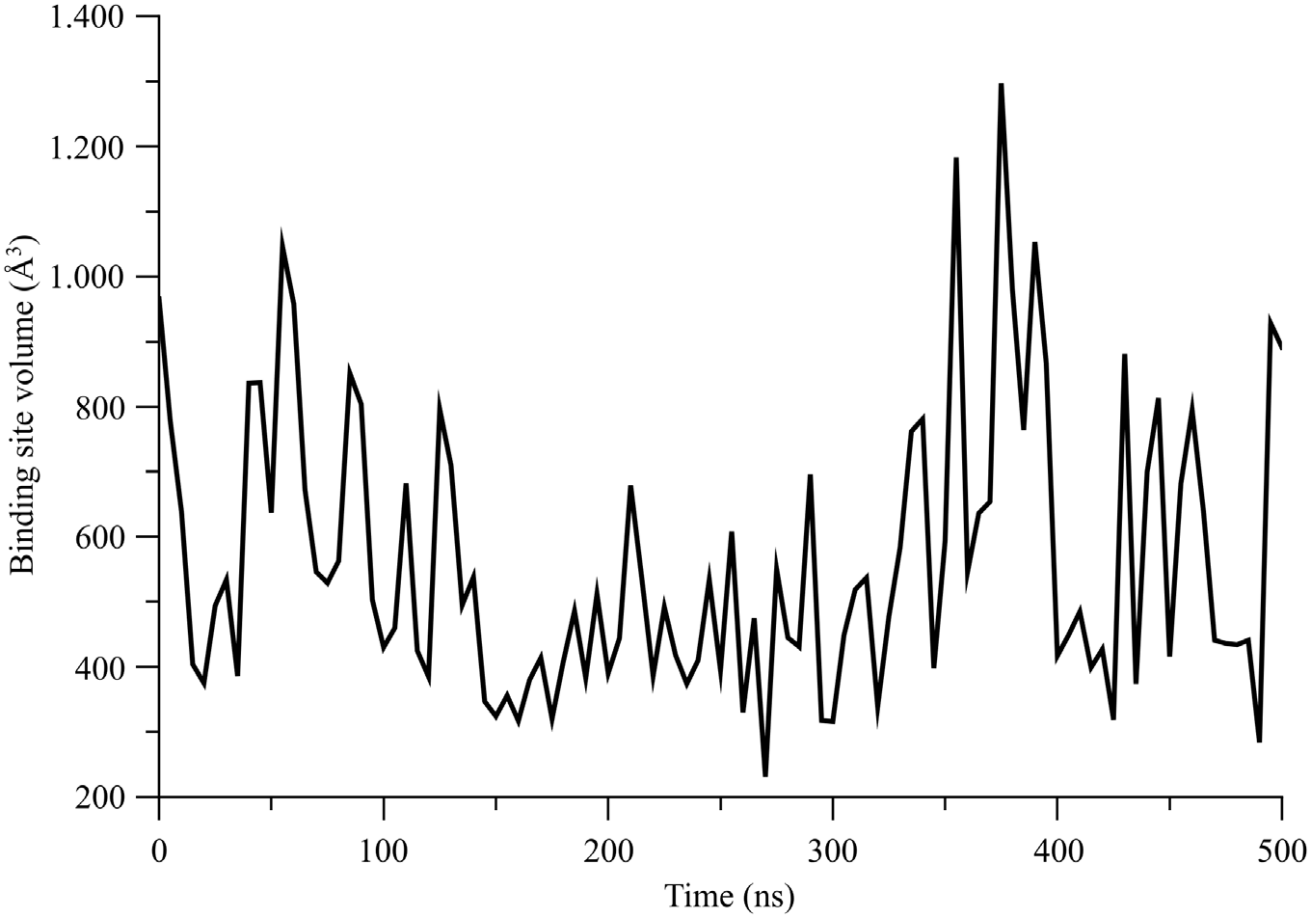
POCASA binding site volume measured every 5 ns of the 0.5 *µ*s all-atom MD simulation of the *µ*OR structure.

**Figure 7:**
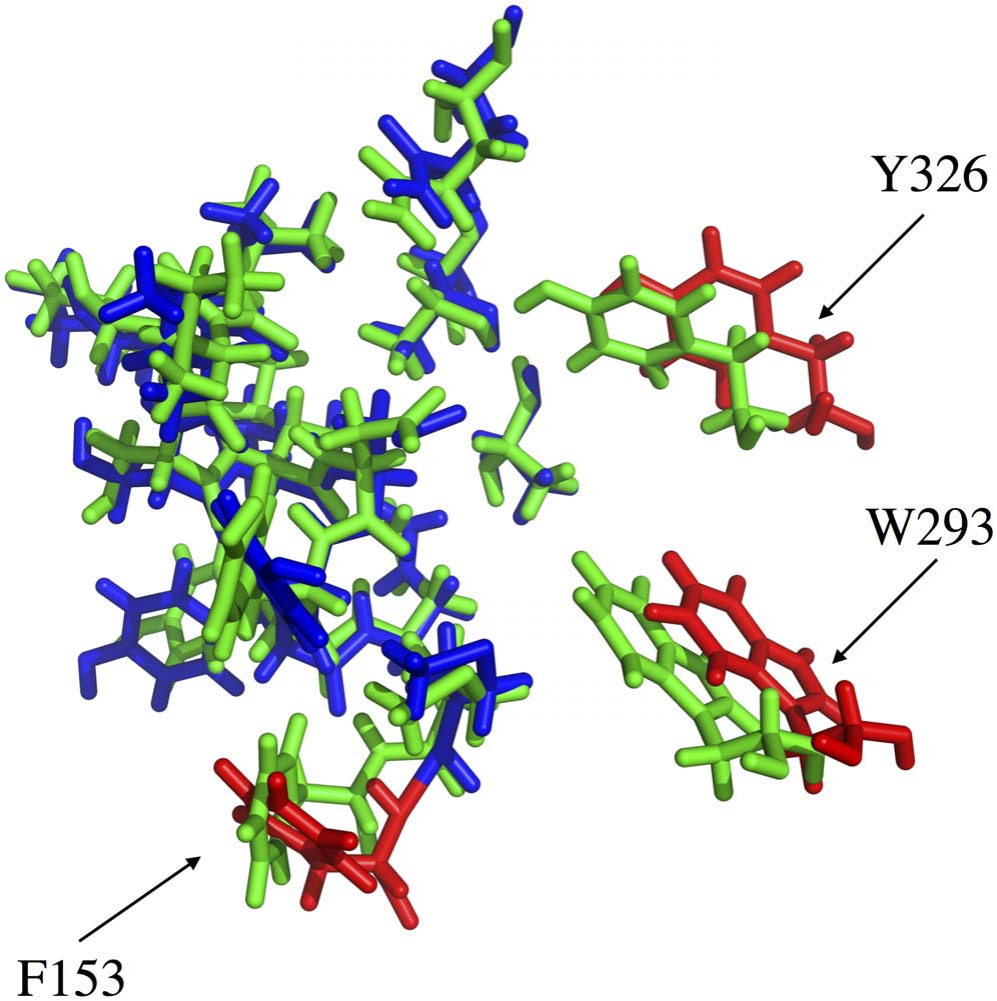
Superimposition of the binding site conformations observed at the 270^th^ (green) and 375^th^ (blue) ns frames, *i.e.*, the frames of the minimum and maximum POCASA volume, respectively, as deserved during the 0.5 *µ*s all-atom MD simulation of the *µ*OR structure. The three residues labelled, *i.e*., F153, W293, and Y326, are the only ones located in the minimum volume pocket. The corresponding residues of the binding site conformation related to the maximum POCASA volume are presented in red.

As described in the *Methods*, we used the POCASA software as a pocket detection algorithm on a series of frames recorded during the µOR MD production run. The analysis was performed each 5 ns of the all-atom 0.5 *µ*s MD trajectory. For every selected frame, we checked that the principal pocket detected indeed corresponds to the expected agonist/antagonist binding site by visualizing if the pocket is located specifically near the residue D147, this last one being well known to interact with several agonists and/or antagonists of µOR [72]. The POCASA volume (PV) computed for each selected frame of the all-atom 0.5 *µ*s MD trajectory is presented in Fig. 6. The observed maximum value is 1297 Å^3^ at 375 ns, the minimum 231 Å^3^ being recorded at 270 ns, revealing how the binding site volume could vary during the simulation. The alignment between the µOR conformations corresponding to these extreme PV values results in a RMSD value of 1.22 Å. This value has to be related to the conformations of specific side chains close to the binding sites. Several side chains could evidently fill the binding site after having changed their conformations, thus hindering the extension of the cavity volume towards the inter-helix space. Fig. 7 illustrates a superimposition of the conformations of the binding site recorded at the 270 and 375^th^ ns frames. In the figure, we focus on residues located at a maximum distance of 5 Å around residue D147. In the 270^th^ ns frame, characterized by the minimum PV value, three additional residues are present, F152 in H3, W293 in H6, and Y326 in H7, as compared to the 375^th^ ns frame with the maximum PV. As the side chains of the additional residues W293 and Y326 are translated towards the cavity of the µOR structure, it is clear why the PV values and also the binding site characteristics can be influenced by the flexibility of the helices. Interestingly, residue F153 is subject to a perpendicular rotation in addition to a translational motion, an important characteristic for an aromatic nature regarding the geometry required for a plausible interaction of this residue with an aromatic moiety of a ligand.

Such a flexibility implies a variation of the inter-helix distances and hence a closure/opening of the binding pocket. Let us also stress that exploring the binding site properties related to the µOR flexibility is very important when one knows that residue W293 has been found to be essential in the binding of some most used anesthetics like fentanyl, carfentanyl, alfentanil, or sufentanil but not for the binding of remifentanyl according to some recent experimental studies [72]. In this study, it is also observed that Y326 in H7 is an important residue for agonists and/or antagonists binding. Y326 is active in the binding of fentanyl, alfentanil or sufentanil but not of carfentanil and remifentanyl.

We also observed that, at the extracellular side of the µOR structure, conformations corresponding to the extreme PV values can be rather different. EL2 is differently localized compared to the rest of the receptor. This loop plays an important role in the opening of the µOR cavity. It affects the available space for µOR ligands of variable volumes. Also, H6 can adopt different conformational states, depicting a plausible closing of the µOR cavity. Indeed, the conformational flexibility of H6 is mainly observed at the extracellular side. We assume that the bendability of H6 originates from the presence of proline P295, in close vicinity to W293, allowing H6 to deviate from its average conformation like a pivot. P295 is normally a highly conserved residue in many GPCRs. It could play a role in the ligand-induced mechanisms allowing GPCRs to “be docked” by ligands of different sizes.

The same situation can be observed for helix H7, for which the residue Y326 is close to proline P333, and which could also act as a pivot to approach Y326 in the binding to the µOR cavity. The high bendability of H6 and H7, the third and the first most bendable helices according to our scale of flexibility, is determined from the BENDIX representations (Table 3). This could be explained in terms of their biological function, bringing some essential residues, *i.e*., W293 in H6 or Y326 in H7, for ligand binding in the µOR cavity thanks to the presence of a proline kink near these residues.

**Figure 8:**
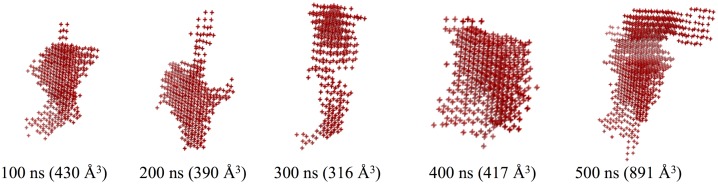
Representation of the pocket binding site as detected with the POCASA software. The shape comes from the gathering of the positions of the POCASA probe sphere explored every 100^th^ ns frame of the 0.5 *µ*s all-atom MD simulation of the *µ*OR structure. The five pockets illustrate the variation of the binding site shape during the simulation.

In Fig. 8, we present the shape of the pocket cavity as gathered with POCASA every 100^th^ ns frame. It can be clearly seen that for the binding site not only the volume matters but also of the geometry. Even if several pockets may have a very similar PV, *i.e.*, a value of 417 Å^3^ for the 400^th^ ns frame *versus* 430 Å^3^ for the 100^th^ ns frame, their topologies may really be different with a rather spherical shape for the 100^th^ ns frame compared to a more extended one for the 400^th^ ns frame.

This plasticity of the binding sites observed during the MD simulation is an explanation of why the µOR could interact with a diversity of ligands exhibiting a large panel of volume sizes. These results demonstrate that taking into account the flexibility properties, and more specifically the helix bendability in the case of GPCRs, is very important for docking studies as they directly influence the residues present in the binding cavity.

The correlations between the motions of the different parts of the µOR structure and potent explanations that could facilitate early ligand binding events are examined in the following section.

### D. Functional significance behind the µOR flexibility

#### D.1 GeoStaS: A method to modularize the µOR structure

The GeoStaS algorithm, described in Methods, was applied to the all-atom MD results in order to subdivide the *µ*OR into dynamical domains. The first stage was to determine the number of domains in the *µ*OR structure using the strategy of Shao *et al.* [73]. The distance between merged clusters, called the critical distance (CD), was thus computed at each clustering iteration (S4 Fig.). The resulting plot allows determining the number of domains in *µ*OR by selecting the coordinate at which the CD strongly differs from its previous value. The obtained optimal number of domains was seven.

**Figure 9:**
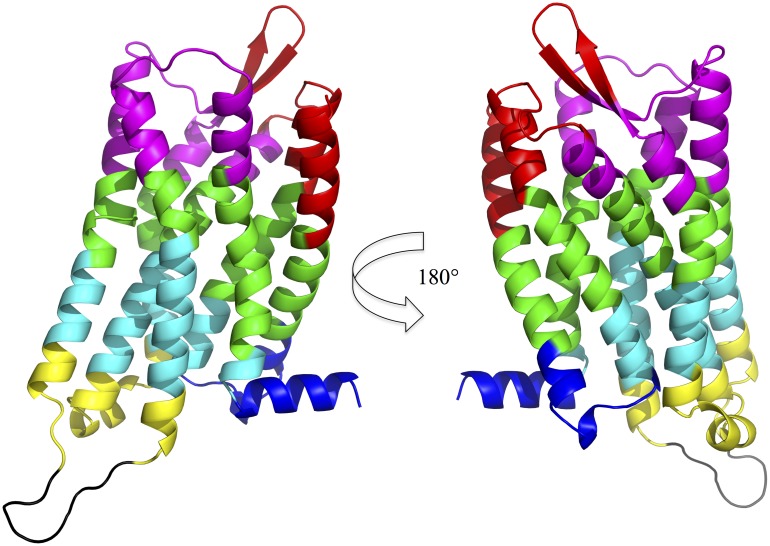
Domain subdivisions of the *µ*OR structure as obtained using the GeoStaS algorithm applied to the 0.5 *µ*s all-atom MD simulation. The seven resulting domains are D1 (red), D2 (green), D3 (blue), D4 (cyan), D5 (pink), D6 (yellow), and D7 (black).

In Fig. 9, we illustrate the seven dynamical domains of the *µ*OR structure as obtained from GeoStaS. The composition of the domains in terms of helices, ILs, and ELs, as well as the average RMSF per residue for each domain are presented in Table 4. For the 288 residues of *µ*OR, the dynamical domains gather very different number of residues, *i.e.*, 38, 74, 24, 54, 57, 31, or only 10 residues. We also noted that none of the domains correspond to a block of one full helix but are rather constituted of sets of residues belonging to different helices or to ILs/ELs loops, disclosing a high natural intercommunication or an interaction aptitude between sub-domains of *µ*OR. Among the three most populated dynamical domains, *i.e.*, D2, D5, and D4, two of them, *i.e.*, D2 and D4, are constituted of residues belonging only to helices. Moreover, D2 is the only dynamical domain containing residues from each of the helices of the bundle of *µ*OR. D4 contains also residues from each of the helices, except H1. Let us also mention that D2 and D4 are localized by the center of the receptor axis. The obtention of these two domains suggests that the flexibility of the *µ*OR center is essentially summarized in a pair of two blocks that are uncoupled from the dynamics of the rest of the receptor. The two lower values of the average RMSF per domain, 0.73 and 0.65 Å, respectively, correspond to those D2 and D4 domains, illustrating that they are the most rigid parts of the *µ*OR structure. Moreover, these two domains are, along with D5, the most populated domains in term of residues, *i.e.,* with 74 and 54 residues, respectively.

**Table 4:** Numbers of residues in the helices (H1–H8), ILs, and ELs composing the different domains, *i.e.*, D1 to D7, as determined with the GeoStaS algorithm applied to the 0.5 *µ*s all-atom *µ*OR MD simulation.

Domain	H1	H2	H3	H4	H5	H6	H7	H8	EL1	EL2	EL3	IL1	IL2	IL3	RMSF (Å)
D1 [38]	13	9	1						6	9					1.42
D2 [74]	13	12	9	8	10	6	16								0.73
D3 [24]	5	1						12	6						0.94
D4 [54]		7	13	8	8	14	4								0.65
D5 [57]			7	6	11		16			10	7				1.15
D6 [31]			4	3	5	5							10	4	1.14
D7 [10]														10	2.57

Numbers between brackets correspond to the total number of residues gathered in a domain. The last column reports the mean Root Mean Square Fluctuation (RMSF) per residue for each domain. The RMSF values were computed over all C*α* atoms of the *µ*OR.

The extracellular side of *µ*OR also involves two dynamical domains, *i.e*., D5 and D1. They are among the most flexible parts of the *µ*OR structure according to the values of their average RMSF per residue value, 1.42 and 1.15 Å, respectively. Finally, the three least populated dynamical domains in terms of the number of residues in *µ*OR, *i.e.*, D3, D6, and D7, are located in the intracellular part. Interestingly, D7 mainly represents the longer IL of the receptor, *i.e.*, IL3, illustrating its possible role as an independent module and implicated as a recruiter in the recognition of several proteins as reported in literature [74]. Moreover, IL3 is clearly the most flexible compared to the other parts of the receptor structure according to its mean RMSF per residue value, *i.e.*, 2.57 Å. D3, with a residue composition consisting of the entire H8 and the intracellular side of H1, can play a role in the recognition of µOR with a G-protein. H8 is the place where the G-protein/µOR interactions occur whereas the intracellular section of H1 can be coupled to helix H8 to leave space for the trimeric G-protein.

Such a subdivision of the µOR structure is directly related to the allosteric nature of the µOR, *i.e.*, a set of flexible blocks divided into three regions. The first region, composed of two modules in the extracellular side of µOR, plays a plausible role in the opening/closing of the structure during the early events of ligand recognition. The two larger modules in the center are involved in the transduction of the ligand-induced mechanism to the extracellular side. The last one contains three modules; among them D3 acting as the transmitter to the G-protein of ligand messaging and finally the independent module D7 acts like an arm to interact with a great variety of proteins. The mean RMSF per residue values support our hypothesis of a more rigid central domain compared to the intra- and extracellular domains, with a highly flexible D7 module compared to the other parts of µOR.

#### D.2 A network-based approach to partition the µOR structure

A complementary approach, *i.e.*, the residue-residue network approach as described in *Methods*, was applied to modularize the µOR structure. To construct the network of residue-residue interactions, each residue was assigned to only one node. The method was applied to a coarse-grained representation of the µOR system where each bead is centered on one C*α* atoms. Edges of the network were determined by considering the dynamical information extracted from the MD results, *i.e.*, a correlation matrix of distances between pairs of beads as explained in Methods. The nodes were connected together only if they were within a distance of 4.5 Å, for a minimum time of 75% of the MD production trajectory. Edges between neighboring C*α* atoms along the primary sequence were disregarded to avoid any bias due to non-relevant correlated motions. The network contained 1936 edges involving 288 nodes, each node corresponding to one residue. The number of edges and the mean number of edges per residue for the helices, ILs, and ELs of µOR are presented in Table 5.

**Table 5:** Number of edges and its mean value per residue in the residue-residue interaction network determined from the all-atom 0.5 *µ*s MD simulation for the helices, ILs, and ELs of the *µ*OR structure.

Element	Total of edges	Mean number of edges	Element	Total of edges	Average edge
H1	217	7	H8	77	6.4
H2	232	8	EL1	33	5.5
H3	278	8.2	EL2	88	4.6
H4	176	7	EL3	19	3.2
H5	235	6.9	IL1	32	5.3
H6	234	7.1	IL2	54	5.4
H7	198	7.1	IL3	56	4

As seen from Table 5, helices are in general more connected than ILs/ELs with higher numbers of edges. It is reported in literature that H3 could have a potential role in the transduction of the ligand-induced signal. It is thus interesting to note that H3 is the most connected helix with a mean residue connectivity of 8.2. The least connected helix is H8 with a mean connectivity of 6.4 per residue. H8 has to be effectively more independent and free than the other helices in order to recognize a G-protein during the signaling process. Among the three ILs, IL3 is the least connected loop, supporting its potential role as an arm module involved in the recognition of partner proteins of µOR. The importance of the edges in the residue-residue interactions was weighted on the correlation data extracted from the MD simulation as described in *Methods*. The so-obtained network of the µOR structure and its weighted version are given in S5 Fig.

**Figure 10:**
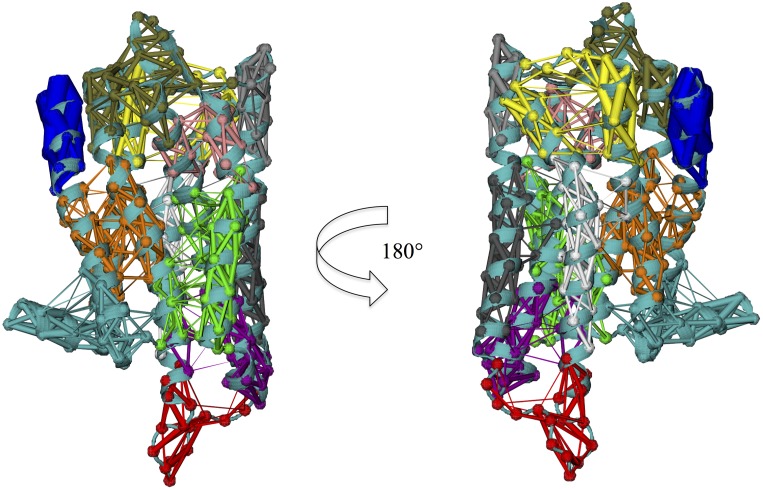
Modularization of the *µ*OR structure as obtained through the network approach applied to the 0.5 *µ*s MD simulation. The twelve resulting modules are M1 (blue), M2 (red), M3 (gray black), M4 (orange), M5 (yellow), M6 (brown), M7 (gray), M8 (green), M9 (white), M10 (pink), M11 (cyan), and M12 (purple).

From the weighted network, we generated the communities to partition the µOR structure. Communities are related to the residues that move together by block as computed from the correlation-based weight network such as explained in *Methods*. Twelve communities were identified, resulting therefore to a least coarse-grained modularization of the µOR structure compared to the GeoStaS approach discussed above. The composition in terms of helix/ELs/ILs of the twelve modules is reported in Table 6. Fig. 10 illustrates the subdivision of the receptor structure into the twelve modules.

**Table 6:** Composition in terms of helices (H1–H8), ILs, and ELs of the different modules, *i.e.*, M1 to M12, as determined through the network modularization of the all-atom 0.5 *µ*s MD simulation. Numbers between brackets correspond to the total number of residues gathered in each of the modules of the *µ*OR structure.

Module	H1	H2	H3	H4	H5	H6	H7	H8	EL1	EL2	EL3	IL1	IL2	IL3
M1 [13]	13													
M2 [17]						3								14
M3 [24]			3		21									
M4 [36]	11	13					12							
M5 [27]						9	12				6			
M6 [33]		9	5									6	13	
M7 [15]					11					4				
M8 [34]		6	8	17								1	2	
M9 [20]			1			17	2							
M10 [17]			7	8						2				
M11 [28]	7	1					3	12				5		
M12 [24]			10		2	4							8	

As for the GeoStaS algorithm, the modularization leads to various domains in terms of helices, ILs, and ELs, which are nevertheless less heterogeneous compared to what is observed with the so-called network due to the least coarse modularization of µOR. Moreover, the distribution of the residue population between the modules of the network approach is naturally less scattered. Hence, the most populated module is M4, composed of 36 residues, and the least populated one is M1 with 13 residues, whereas the most and least populated dynamical domains generated using the GeoStaS algorithm did have 74 and 10 residues, respectively. Therefore, some domains obtained with the modularization approach can almost be assimilated to entire- or half-block of a single helix, IL, or EL. The M1 module corresponds to the upper-half part of H1, consisting of 13 residues of the entire 30 residues of H1. M2, composed of 17 residues, corresponds to the entire 14-residue IL3 loop, the three last residues coming from the extracellular side of H6. Among the 34 residues of H5, 21 coming from its central part are grouped in module M3 whereas 11 other residues of the extracellular section of H5 are located in module M7. H5 is principally found in the M3 and M7 modules, making them weakly heterogeneous composed modules. A similar but still less homogeneous situation is observed for H6 as for a total of 33 residues, 17 are located in module M9 which is composed of 20 residues, with the 16 remaining residues dispersed between three modules, *i.e.*, M2, M5, and M12. The subdivision of the µOR structure with the network approach does however not lead to a one-to-one correspondence between module and secondary structure elements, *i.e*., helix, IL, or EL. Some modules are still very heterogeneous in terms of their helix, IL, and EL content like modules M4, M5, M6, M8, M10, M11, and M12, representing together a number of 199 residues over a total of 288. The heterogeneity in terms of helix composition of several modules supports concomitant motions of parts of diverse helices due to their interactions. As a consequence, the flexibility properties of µOR are disclosing an inter-helix communication related to the allosteric nature of GPCRs and the need to propagate rapidly throughout the membrane domain the activation message due to the presence of a ligand.

#### D.3 Comparison between the GeoStaS and the network-based µOR modularization

The finer-grained modularization of the µOR structure coming from the network approach is actually in good agreement with the coarser model obtained from the GeoStas algorithm. By grouping some modules coming from the network approach, we did recover a similar subdivision as the one obtained with GeoStaS. The GeoStaS dynamical domain D1 corresponds to the two M1 and M6 modules of the network approach, 28 residues are in common among the 38 residues of D1 and the total of 46 residues for both M1 and M6. The M5/M7/M10 modules, grouping together 59 residues, have 49 of them that are identical with the 57 residues of D5. The extracellular part of the µOR structure then corresponds to two GeoStaS domains (or five network-related) modules as the pair of domains D1/D5 was well related to the extracellular side. An interesting connection between the GeoStaS and the network approach is noticed for the central part of µOR: paired domains D2/D4, containing a total of 128 residues, have 102 residues in common with the grouped M3/M4/M8/M9 modules, containing a total of 114 residues.

Regarding the intracellular part of µOR, all of the 10 residues of the D7 domain are comprised in module M2, composed of 17 residues. This section of µOR corresponds to loop IL3, confirming its dynamical independency as a recognition module for partner proteins. In parallel, a good correlation is found between M11 with 28 residues and D3 with 24 ones, for which 22 identical residues constitute the entire H8 and the extracellular part of H1. Finally, M12 with 24 residues and D6 with 31 ones share 15 residues. Both GeoStaS and network methods describe the same modular-related flexibility of µOR, and the GeoStaS modularization is in a way just a coarse-grained version of the network approach. The two approaches thus depict a clear separation between the extracellular side, the central section, and the intracellular part of µOR according to the flexibility properties computed from the 0.5 *µ*s MD simulation.

We thus understand that the flexibility of the extracellular side as a well-defined structural block is related to its role in allowing the docking of a large variety of ligands. The ligands can have very different sizes due to the motion of the EL2 loop and also to the opening/closing of the extracellular sections of the µOR helices. This observation is correlated with the high variation of the volume of the binding site as discussed previously, as well as with the role of the helices H6 and H7 making essential residues available for ligand binding. On the order hand, the µOR central section is a more rigid part, constituting larger domains or modules as obtained with both the GeoStaS and network approaches. The less flexible behavior of the central part of µOR is related to the function of GPCRs. Hence, once a ligand is docked, the signaling process through the membrane requires high communication ability between the helices in order to be effective and to trigger the allosteric conformation change at the extracellular level. Regarding this latter receptor part, the independence of IL3 as a flexible block is also related to the µOR function. IL3 has a well-known role in the interaction mechanisms between µOR and other proteins. A flexibility unit composed of H8 and the extracellular part of H1 is associated with the potential role of H8 in the recognition of a G-protein once the ligand-induced signal is transduced throughout µOR. The fact that the flexibility of the intracellular section of H1 is related to the one of H8 can be understood as a functional requirement to leave space when a G-protein is interacting with µOR.

In the last section, we will focus on the plausible correlated motions of the µOR domains proposed here above in order to understand the conformational engine due to µOR intrinsic flexibility properties.

### E. Insights on the coupling between the µOR domains

We used the Essential Dynamics Analysis (EDA) technique, also known as Principal Components Analysis (PCA), to decipher the plausible correlation between the extracellular, the central, and the intracellular parts, and the functional significance behind the domain-related flexibility properties of the µOR structure.

**Figure 11:**
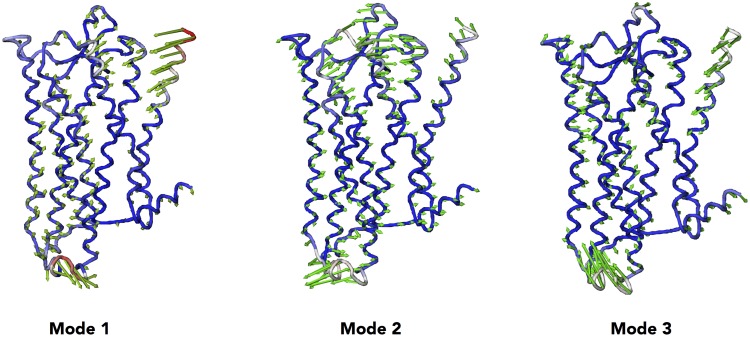
Vector field representation of the three first principal modes obtained from the 0.5 *µ*s all-atom MD simulation of the *µ*OR structure.

EDA was applied to the all-atom 0.5 *µ*s MD run by considering only the coordinates of the C*α* atoms in constructing the covariance matrix. The first 20, 10, and 5 calculated eigenvectors represent 80, 68 and 54%, respectively, of all µOR movements supporting the assumption that the functional dynamics of µOR is dominated by principal collective modes. Among them, the five more preponderant ones have a value of 23, 11, 9, 6, and 5%, respectively. The motions resulting from the three first eigenvectors are presented in Fig. 11.

The first mode depicts motions of the extracellular part of helices H1, H2, H4, H5, H6, H7, and more slightly H3, along with movements of IL2, and especially IL3 in the intracellular part. This first mode is therefore showing a concomitant motion between the extra- and intracellular domains of µOR whereas the central domain is still a very rigid part. The same behavior is observed for the second mode. However, the direction of the extracellular/intracellular motions shows a twist motion linked to the previous observation of the different binding site shapes.

Interestingly, a strong correlation is observed in the third mode between the extracellular side, for which an opening/closing motion of the µOR cavity is observed, along with an elongation motion of helix H8 and a large motion of the H1 extracellular part. Therefore, the third mode supports the allosteric nature of the µOR by illustrating the coupling motions between a closure of the µOR cavity, which can be due to a local ligand-induced fit, and a subsequent motion of the H8 surrounding needed to recognize a G-protein.

The EDA analysis of the µOR all-atom MD simulation supports the hypothesis that the flexibility properties of GPCRs are directly linked to their functions, *i.e.*, the transduction of intracellular message throughout interactions with partner proteins like G-proteins as well as other partners throughout the flexibility as a module of the intracellular loops, as especially the IL3 one in the case of µOR.

The rigidity of the central part of µOR is also well related to the GPCR function, as a weak flexibility of the center of a GPCR allows a better communication between the helices to rapidly transduce the ligand-induced message in the extracellular part.

### Conclusion

Deciphering the flexibility properties of proteins is one of the current challenges in molecular modeling. In this study, we have investigated the flexibility properties of the *µ*OR structure at the ligand-free state in a bilayer environment by performing a sub-microsecond MD simulation.

We first showed that the overall shape of *µ*OR is highly conserved. However, the bendability scale of its helices has a clear influence on the conformational state of its binding sites. More specifically, we rationalized the proximity between proline and the essential residues to ligand binding. The proline residues induce a kink in the *µ*OR helices in order to bring together closer the binding residues, especially in helices H6 and H7.

The study of the *µ*OR binding site dynamics indicated that our MD studies can provide a diversity of conformations of the binding site leading to a wide variety of shapes and volume values. As a consequence, MD is not only a valuable technique to generate alternative conformations of a receptor but also a way to study specific local deformations of the *µ*OR binding site. Our study also suggests a high plasticity of the *µ*OR binding site, exhibiting several different shapes with a high volume variation, accounting for the experimentally known ability of *µ*OR to bind ligands of different sizes.

Next, we illustrated that the flexibility of *µ*OR can be decomposed into a few domains by applying two different algorithms on the dynamical information extracted from the all-atom MD simulation. The partitioning of the *µ*OR structure consists of a few hubs in its intra-, extracellular, and membrane parts. Such a subdivision demonstrates the *µ*OR ability, as for other GPCRs, to articulate modules together, a mechanical feature of the GPCR folding that could explain the rapid transmission of cellular message. Essential Dynamics Analysis has moreover given a functional significance behind the *µ*OR dynamics as correlated motions between intra- and extracellular sections of *µ*OR have been depicted, while the central *µ*OR parts remain very rigid.

As a conclusion, the flexibility properties of *µ*OR predispose to a coupling between distant domains, which is the motor behind the allosteric behavior and the ligand-induced conformational changes. The highly rigid modules of the *µ*OR central part being the mediators to communicate the message of the ligand presence. The intrinsic flexibility of the *µ*OR could be also one of the explanations of the ability of this receptor to present a significant basal activity. For this purpose, long MD simulations, *i.e.*, micro- to milli-second timescale, would be valuable to observe a certain active form of *µ*OR.

Our approach to study the *µ*OR flexibility in the apo-form thus contributes to the understanding of the dynamical modes of GPCRs and their biological implications. As a perspective, we propose that, prior to ligand docking simulations, alternative conformations of the binding site need to be generated as we clearly showed how its shape and volume can vary significantly. The techniques we used to characterize the binding site characteristics could then be applied to classify its alternative conformations in a library of conformations. Another perspective is to apply the strategy developed here to perform MD simulations of several GPCRs in the apo-form. It would supplement the more usual approach of investigating GPCRs dynamics in their liganted form. Such simulations could elucidate how the major flexibility properties of GPCRs influence ligand binding and the characteristics of their binding site, but also more complex mechanisms such as GPCRs oligomerization or, more generally, interactions with partner proteins. MD simulations of other GPCRs should therefore provide structural information about the basal activity of GPCRs.

## Supporting Information

S1 Fig Top: Root Mean Square Deviation (RMSD) between the starting *µ*OR structure and its subsequent conformations generated during the 25 ns MD equilibration protocol. RMSD values were computed on all the heavy atoms of *µ*OR. Grey strips illustrate large RMSD fluctuations. Bottom left: Mean value of the RMSD between the starting *µ*OR structure and its subsequent conformations on an increased length simulation windows with a step of 1 ns. RMSD values were computed over all heavy atoms of *µ*OR. Bottom right: Standard deviation of the RMSD between the starting *µ*OR structure and its subsequent conformations on an increased length simulation windows with a step of 1 ns. RMSD values were computed over all heavy atoms of *µ*OR.
(TIFF)

S2 Fig **Ramachandran plot of the last nanosecond averaged structures of the** ***µ*** **OR structure generated during the 25 ns MD equilibration protocol.** It indicates that 98.5% of the residues are located in the most favorable regions in blue according to the φ and ψ angles. Orange points are related to the remaining 1.5% of residues located in the lighter blue allowed regions of the Ramachandran plot.
(TIFF)

S3 Fig **Root Mean Square Deviation (RMSD) between the starting** ***µ*** **OR structure and its subsequent conformations generated during the MD simulation, including the 25 ns equilibration stage and the 0.5** ***µ*** **s production trajectory.** RMSD values were computed over all heavy atoms of *µ*OR.
(TIFF)

S4 Fig **Critical distance evolution in function of the clustering steps as implemented in the GeoStaS algorithm, with focus on the red region of the principal graph to point out the chosen number of clustering steps,** ***i.e*** **., seven.** The GeoStaS algorithm was applied on the all-atom 0.5 *µ*s MD simulation of the *µ*OR structure.
(TIFF)

S5 Fig Left: Unweighted and right: Weighted versions of the *µ*OR residue-residue interactions network determined from the all-atom 0.5 *µ*s MD simulation of the *µ*OR structure. Edges between residues, represented as red sphere, are depicted and superimposed on the *µ*OR structure in green. Low and high weighted edges are pointed out to apprehend how the residue-residue interaction network is evolving by taking account the weighted edges.
(TIFF)

S1 Text (DOC)
