# Supplementary figures and images for: On the Modularity of the Intrinsic Flexibility of the µ Opioid Receptor: A Computational Study
Source: PLoS One. 2014 Dec 30;9(12):e115856. doi: 10.1371/journal.pone.0115856 (PMC4280117; doi:10.1371/journal.pone.0115856)

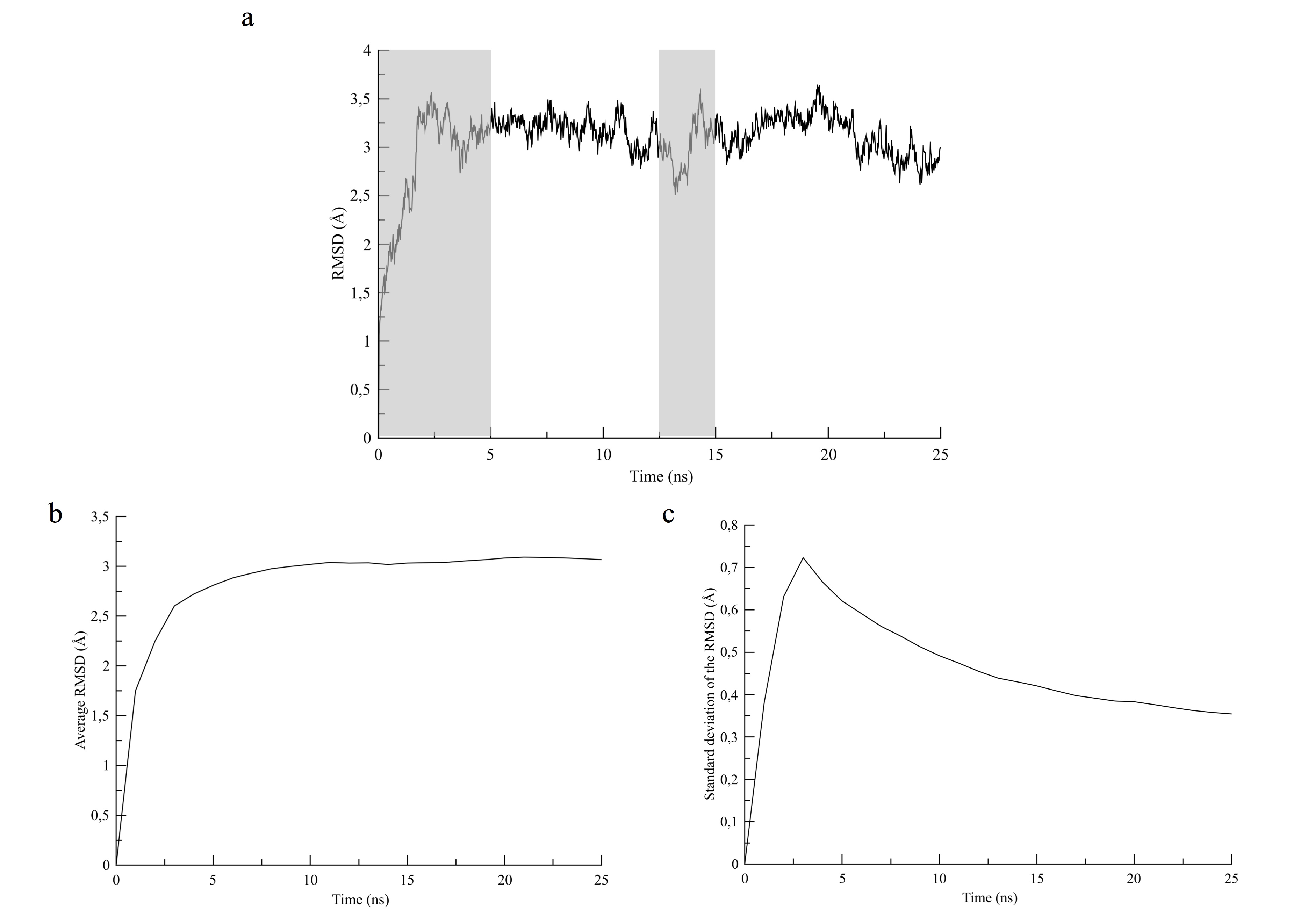

Supplement: S1 Fig — Top: Root Mean Square Deviation (RMSD) between the starting µOR structure and its subsequent conformations generated during the 25 ns MD equilibration protocol. RMSD values were computed on all the heavy atoms of µOR. Grey strips illustrate large RMSD fluctuations. Bottom left: Mean value of the RMSD between the starting µOR structure and its subsequent conformations on an increased length simulation windows with a step of 1 ns. RMSD values were computed over all heavy atoms of µOR. Bottom right: Standard deviation of the RMSD between the starting µOR structure and its subsequent conformations on an increased length simulation windows with a step of 1 ns. RMSD values were computed over all heavy atoms of µOR. (TIFF) [file pone.0115856.s001.tiff]

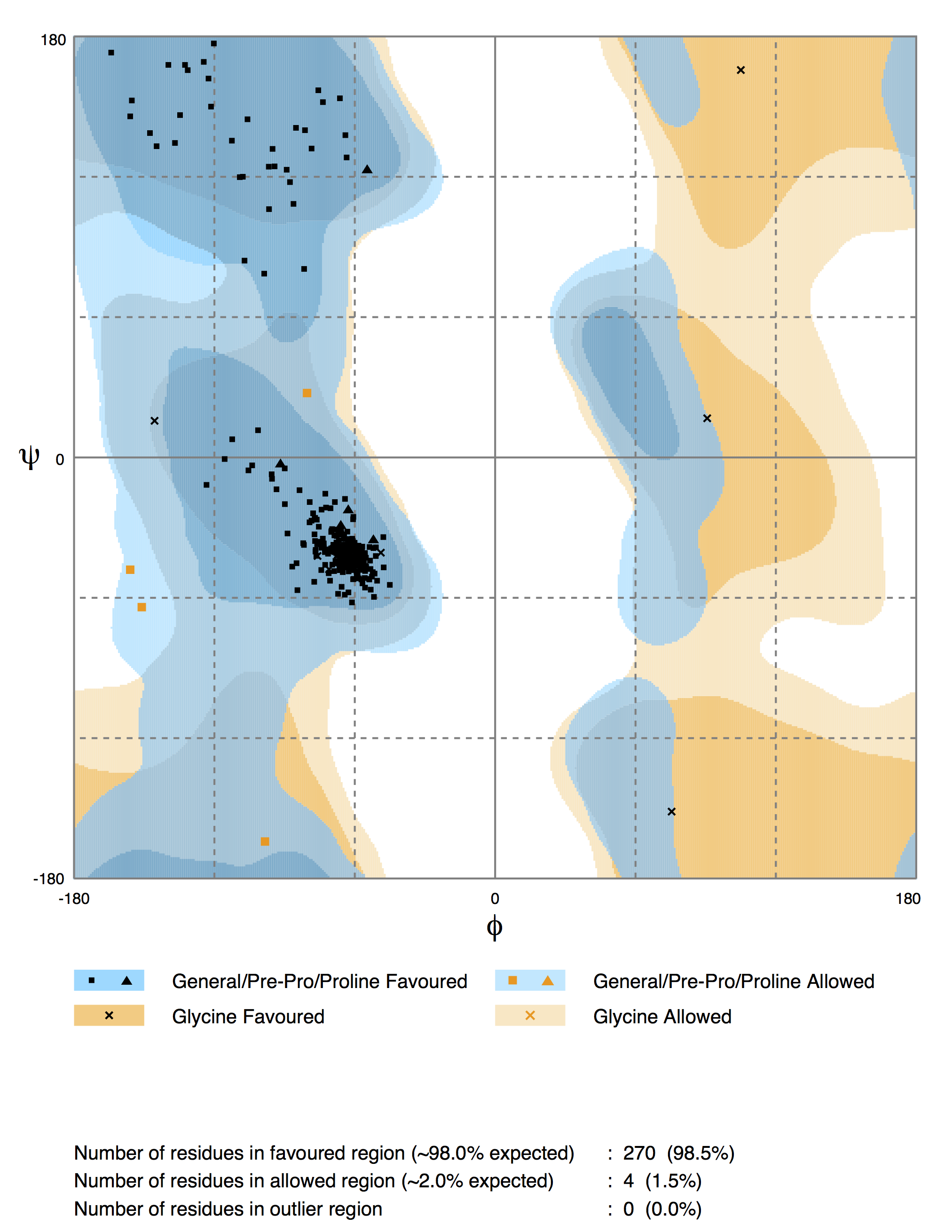

Supplement: S2 Fig — Ramachandran plot of the last nanosecond averaged structures of the µ OR structure generated during the 25 ns MD equilibration protocol. It indicates that 98.5% of the residues are located in the most favorable regions in blue according to the φ and ψ angles. Orange points are related to the remaining 1.5% of residues located in the lighter blue allowed regions of the Ramachandran plot. (TIFF) [file pone.0115856.s002.tiff]

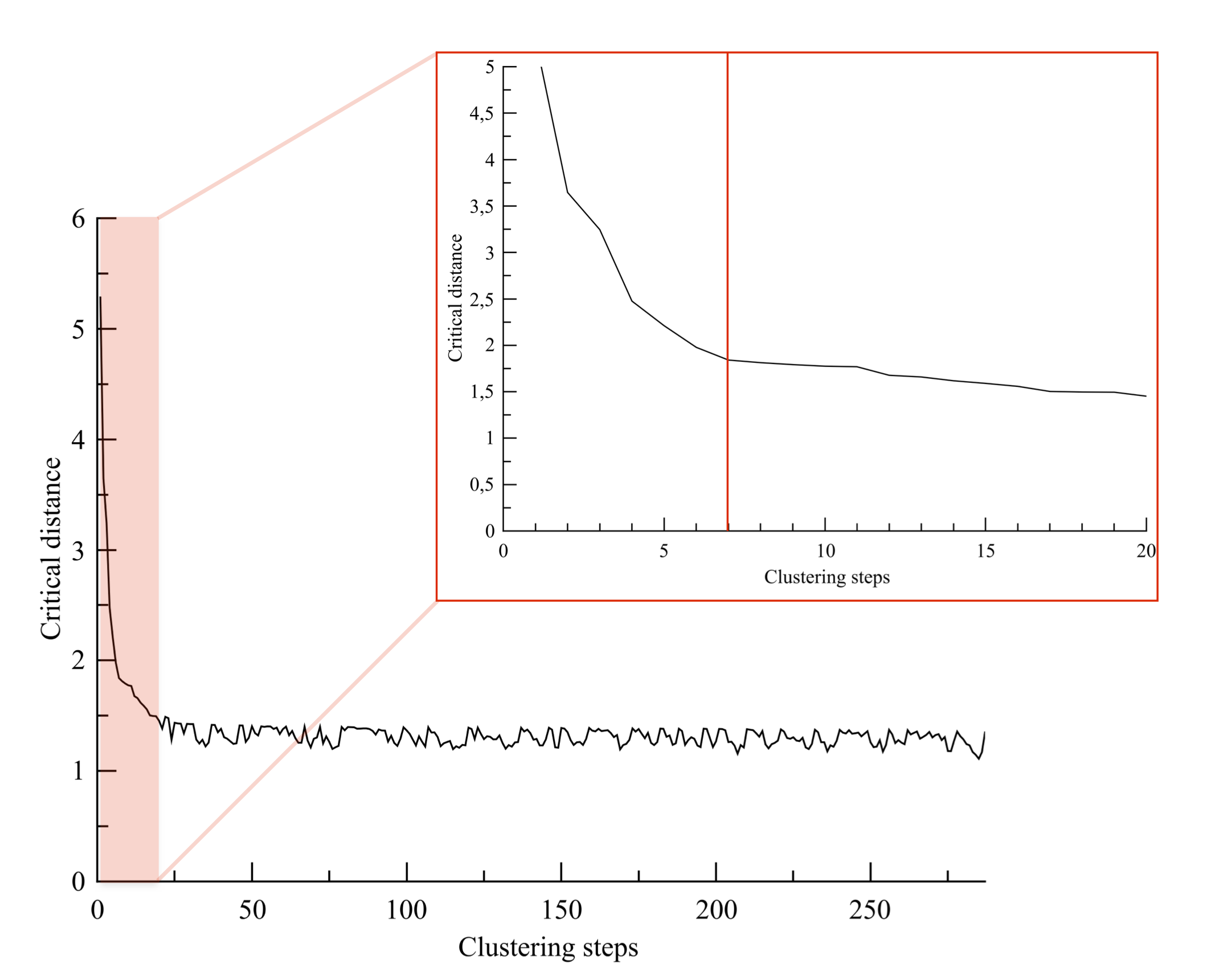

Supplement: S4 Fig — Critical distance evolution in function of the clustering steps as implemented in the GeoStaS algorithm, with focus on the red region of the principal graph to point out the chosen number of clustering steps, i.e ., seven. The GeoStaS algorithm was applied on the all-atom 0.5 µs MD simulation of the µOR structure. (TIFF) [file pone.0115856.s004.tiff]

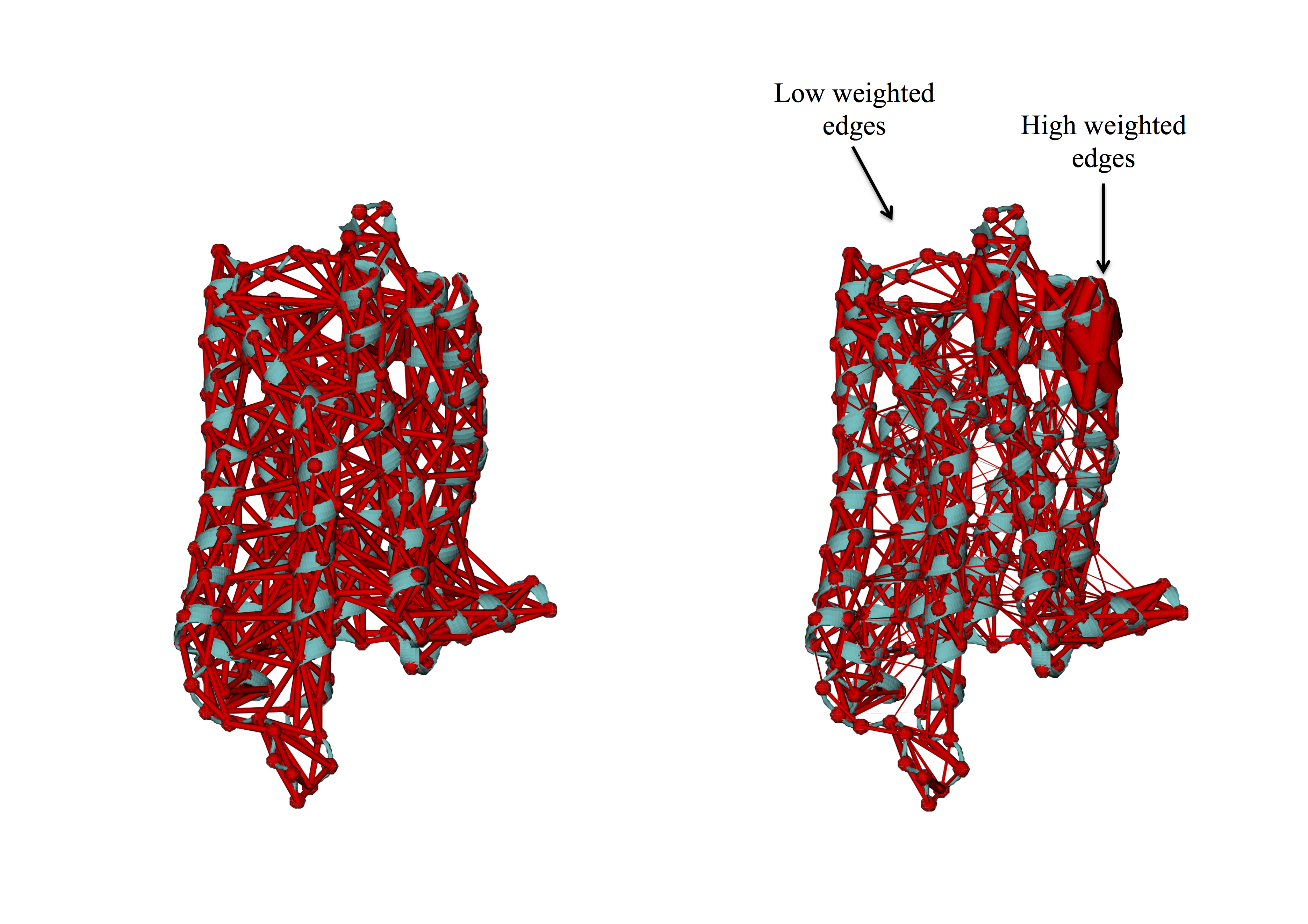

Supplement: S5 Fig — Left: Unweighted and right: Weighted versions of the µOR residue-residue interactions network determined from the all-atom 0.5 µs MD simulation of the µOR structure. Edges between residues, represented as red sphere, are depicted and superimposed on the µOR structure in green. Low and high weighted edges are pointed out to apprehend how the residue-residue interaction network is evolving by taking account the weighted edges. (TIFF) [file pone.0115856.s005.tiff]
