# Supplementary material for: On the Modularity of the Intrinsic Flexibility of the µ Opioid Receptor: A Computational Study
Source: PLoS One. 2014 Dec 30;9(12):e115856. doi: 10.1371/journal.pone.0115856 (PMC4280117; doi:10.1371/journal.pone.0115856)
Supplement: S1 Text — (DOC) [file pone.0115856.s006.doc]

**Text S1. Validation of the equilibration protocol**

The stability of the *µ*OR structure in a lipid bilayer environment, a required step to remove crystal type constraints, and the optimization of the (re)constructed missing IL3 loop were assessed as follows. We calculated the RMSD between the starting structure of the *µ*OR model and its subsequent conformations along the MD equilibration protocol as described in *Methods*. The RMSD values were calculated over all the heavy atoms of the *µ*OR structure (Fig. S1). After an initial increase of the RMSD values during the first five nanoseconds (ns), the values become smaller (Fig. S1 top). Still, large fluctuations can be encountered, *e.g.*, between 13 and 15 ns, but they are small relative to the size of the system, *i.e.*, 4,728 atoms for *µ*OR. In addition, the mean RMSD and its standard deviation (SD) on a progressively larger simulation window length along the equilibration trajectory, *i.e.*, with a cumulative step of 1 ns, confirms both that the RMSD values reach a plateau (Fig. S1 bottom).

The φ and ψ torsional angles of the receptor backbone were also calculated for the averaged structure determined from the last nanosecond of the 25 ns equilibration phase. The resulting Ramachandran map (Fig. S2) shows that 98.5 % of the residues are in the most favorable regions of φ and ψ angle values usually encountered in proteins. The remaining 1.5 % of the residues are located within the allowed region. Hence, the convergence of the RMSD and the quality assessment through the Ramachandran map of the last structures of the MD equilibration protocol both indicate that the *µ*OR structure considered is reliable and well stabilized in the lipid bilayer environment. We thus relied on the next 0.5 *µ*s production protocol, as described in *Methods*, to further investigate the dynamical behavior of *µ*OR.
